# Examining the association between rehabilitation participation and cognitive and affective states in adult day service users

**DOI:** 10.1002/alz70858_105588

**Published:** 2025-12-26

**Authors:** Naofumi Tanaka, Yoshitaka Ouchi, Yin‐Jung Wang, Masashi Kasuya

**Affiliations:** ^1^ Saitama Medical University International Medical Center, Hidaka, Saitama, Japan; ^2^ Medical corporation Jinsenkai, Sendai, Miyagi, Japan; ^3^ Tohoku Fukushi University, Sendai, Miyagi, Japan; ^4^ Miyagi University, Daiwa, Miyagi, Japan

## Abstract

**Background:**

Day care services support older adults with dementia and their caregivers, providing rehabilitation to maintain and improve the physical function and social participation of the older adults with dementia.

**Method:**

The participants were 224 older adults living in the community who used day‐care services. Users with MMSE scores less than 10 evaluation were excluded. Rehabilitation participation (RP) was measured using the Pittsburgh Rehabilitation Participation Scale (PRPS), and the average score over three consecutive days was used for analysis. Based on the average PRPS scores, the participants were divided into two groups: good RP (>4, *n* = 131) and poor RP (≤4, *n* = 93) groups. Other assessments were the Geriatric depression scale‐15 (GDS‐15) and the Apathy evaluation scale (AES). MMSE, GDS, and AES scores were compared between the groups using Mann‐Whitney U test.

**Result:**

The age of the participants was 85 ± 8 years and 64/160 were men/women. The MMSE scores of the good RP group were significantly larger than those of the poor RP group (*p* <0.02). The GDS scores were not significantly different between the two groups. The AES scores of the good RP group were significantly smaller than those of the poor RP group (*p* <0.01). When further divided into three groups according to MMSE score (≥24, 23‐20 and 19‐10), the good RP group with an MMSE score of 23‐20 had a significantly lower AES score than the poor RP group with the same MMSE score (*p* <0.04). In the other two subgroups, there was no significant difference in the AES scores between the good and poor RP groups.

**Conclusion:**

Rehabilitation participation among older day care users was not associated with depression, but with cognitive impairment and apathy. Furthermore, a significant association between rehabilitation participation and apathy was found in those with mild cognitive impairment. Therefore, an approach targeting apathy would be expected to improve rehabilitation participation status.

